# Sustainability, Eco-Point and Engineering Performance of Different Workability OPC Fly-Ash Mortar Mixes

**DOI:** 10.3390/ma9050341

**Published:** 2016-05-06

**Authors:** Putri Zulaiha Razi, Hashim Abdul Razak, Nur Hafizah A. Khalid

**Affiliations:** 1 StrucHMRS Group, Department of Civil Engineering, University of Malaya, 50603 Kuala Lumpur, Malaysia; putrizulaiha@siswa.um.edu.my; 2 UTM Construction Research Centre (UTM CRC), Institute for Smart Infrastructure and Innovative Construction (ISIIC), Faculty of Civil Engineering, Universiti Teknologi Malaysia, 81310 Johor Bahru, Malaysia; nurhafizah.abdkhalid@gmail.com; 3 Faculty of Civil and Environment Engineering, University of Tun Hussein Onn Malaysia, 86400 Batu Pahat, Malaysia

**Keywords:** engineering performance, environmental performance, CO_2_ footprint, optimum mix, eco-point

## Abstract

This study investigates the engineering performance and CO_2_ footprint of mortar mixers by replacing Portland cement with 10%, 20%, 40% and 60% fly ash, a common industrial waste material. Samples of self-compacting mortar (SCM) were prepared with four different water/binder ratios and varying dosages of superplasticizer to give three ranges of workability, *i.e.*, normal, high and self-compacting mortar mix. The engineering performance was assessed in term of compressive strength after designated curing periods for all mixes. CO_2_ footprint was the environmental impact indicator of each production stage. The optimum mix obtained was at 10% replacement rate for all mixes. Total production emission reduced by 56% when the fly ash replacement rate increased from 0% to 60% (maximum). This is translated to a reduction of 80% in eco-points (assuming that the energy consumption rate of production with 0% fly ash is at 100%). Such re-utilization is encouraged since it is able to reduce possible soil toxicity due to sulfur leaching by 5% to 27% and landfill area by 15% to 91% on average.

## 1. Introduction

Self-compacting mortar (SCM) is an integral part of a self-compacting concrete (SCC) design, a technological evolution of the conventional concrete that does not require any vibrator for compaction. The self-compacting ability of fresh concrete enables it to fill formwork and encapsulate reinforcing bars through the action of gravity while maintaining homogeneity [1]. According to The International Union of Laboratories and Experts in Construction Materials, Systems and Structures (RILEM) [1], the self-compaction improves productivity and the condition of the working environment, as well as enhances the homogeneity of the mixes. The quality of SCC is controlled by three properties—flowing ability, passing ability and resistance to segregation. Flowing ability is the ability to fill the formwork. Having sufficient passing ability allows the mortar to pass through congested reinforcement without any separation or blocking. Lastly, resistance to segregation controls the retaining of coarse components of the mixes to maintain a homogenous state [2,3]. Self-compacting concrete can be used in most applications where normal concrete is commonly used. Nevertheless, its superior performance is more obvious in densely-reinforced structures, though less demanding applications, like backfill, are equally possible. SCC can be fiber reinforced and can be used in both *in situ* construction and pre-cast concrete [1].

In addition, a higher content of fine particles and well-graded aggregate tends to improve the flowability and stability of SCC and SCM [4]. SCC loaded with well-graded aggregates and fine cement produces a dense interfacial transition zone between the aggregate and the cement matrix, which enhances concrete strength and durability [2]. The ability to self-compact has made SCC an attractive choice internationally, but its higher manufacturing cost than conventional concrete has hindered its application in general construction.

Mortars with different binder types have been used since ancient times for different purposes [5]. SCM is preferred due to its ease of mixing while having excellent flowing ability [6]. This reduces casting costs and, again, produces a more homogeneous product. Many studies have been done on SCM [6,7,8,9,10,11,12,13], some focusing on lowering the production cost [14].

The technology may also convert a variety of waste materials into useful by-products as a method to reduce greenhouse gas emissions while solving the problem of the high disposal cost of waste materials, such as fly ash. Notable recent research incorporating fly ash as mineral admixtures, particularly in the SCC, include Bentz *et al.* [15], Dehwah [16], Gesoĝlu *et al.* [17], Bouzoubaa *et al.* [18] Sabet *et al.* [19] and Siddique [20]. Some researchers have also attempted to measure the CO_2_ footprint in concrete. Putri *et al.* [21] studied the effect of fly-ash on the environmental sustainability and engineering performance of OPC-mortar. Flower *et al.* [22] reported the quantification of CO_2_ emissions associated with the manufacture and placement of concrete in the Australian context; the life cycle inventory data were gathered from quarries, batching plants and a few other known sources. Equivalent CO_2_ emissions were presented as the final result, and the potential of fly ash and ground granulated blast furnace slag (GGBS) to reduce the emissions was also investigated and presented. Yang *et al.* [23] proposed an evaluation procedure from cradle to pre-construction by using an individual integration that included the material, production, curing and transportation phases for estimating CO_2_ reduction in alkali-activated concrete (AAC). The performance efficiency indicators for the binder were also determined for different types of concrete. In their findings, the reduction rate of CO_2_ emission of AAC relative to Ordinary Portland Cement (OPC) concrete ranged between 55% and 75%. Cyr *et al.* [24] published an article concerning the design of eco-efficient grouts intended for soil nailing.

The utilization of waste material in cement mortar promotes green technology. Waste materials, such as fly ash, that are deposited into landfills without further consumption consume more space than necessary, not mentioning that they bring adverse environmental impacts, such as leaching and groundwater contamination. As such, green technologies become essential and should be promoted, such as the SCC. This technology, pioneered by Japan, has become the center of attention in recycling fly ash to lessen the CO_2_ footprint. The CO_2_ footprint of mortar production was thus measured in this study, as well as its effects on engineering performance and environmental sustainability. In fact, several authors [25,26,27,28,29] have attempted to relate environmental sustainability from other aspects by utilizing waste materials as supplementary cementing materials where the zero burden hypotheses (co-combustion of fly ashes) were implemented.

## 2. Experimental Program

The mix proportion of the mortar mixes is shown in Table 1 and Table 2. Four different water/binder ratios (0.35, 0.40, 0.45 and 0.50), one control and four replacement levels of fly ash (10%, 20%, 40% and 60%) by weight of cement were adopted for this study. The superplasticizer dosage was determined earlier from the trial mixes to give three ranges of workability, which were normal slump flow (targeted at ≤100 mm), high slump flow (targeted at 150–170 mm) and self-compacting flow (targeted at 240–260 mm). Sixty mortar mixes were prepared to determine the effect of different superplasticizer dosage on the flowability of mortar.

**Figure 1:**
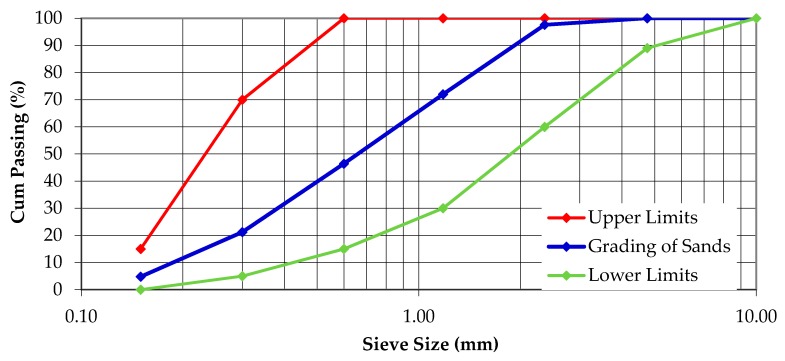
Particle size distribution of silica sand.

This study used an ordinary Portland cement produced from the northern part of Peninsular Malaysia that conforms to the ASTM Type I standard. The silica sand was produced by a local mineral quarry, and the sizes used were 8/16 (1.2–2.4 mm), 16/30 (0.6–1.2 mm), 30/60 (0.3–0.6 mm) and 50/100 (0.3–0.075 mm). A sieve analysis was carried out to ascertaining the fineness modulus of the silica sand used, which was recorded at 2.58 and with a specific gravity of 2.64. The grading of silica sand was based on BS 882 [30], as shown in Figure 1.

The Class F fly ash mineral admixture was obtained locally from a power station located in the northern part of Peninsular Malaysia. The cement and fly ash were analyzed using X-ray fluorescence (XRF) for chemical compositions, and the results together with the physical properties are presented in Table 3. In this present study, the tap water used was checked for any visible contaminants before mixing. The chemical admixture utilized was a modified poly-carboxylate superplasticizer admixture, which had a specific gravity of 1.08.

### 2.1. Specimen Preparation

The test specimens were prepared using a standard steel cube mold of 50 mm × 50 mm × 50 mm in dimensions. Sixty mortar cubes were cast for each mortar mix, giving a total of 1800 test cubes. Prior to casting, the cube molds were cleaned with a soft brush, and a thin layer of oil was applied to facilitate the removal of hardened concrete cube thereafter. A mini slump flow test and mini V-funnel test were conducted to assess the workability of the fresh mortar. Specimens were then cast in the molds, and only the normal and high slump flow mixes were compacted by tamping 25 times uniformly over the cross-section with a steel rod, as specified by ASTM C143/C143M [31]. The mortar specimens were kept covered and cured in the molds for 24 h, after which they were removed from the molds and placed in a curing tank at 20–21 °C, as specified by ASTM C511 [32].

### 2.2. Sample Characterization

The samples were characterized into three different degrees of workability, namely normal flow (targeted at a slump flow diameter of 10–20 mm), high flow (targeted at a slump flow diameter of 150–170 mm) and a designed slump flow diameter range of 240–260 mm specifically for SCM. The targeted flowability was achieved by adjusting the dosage of the modified poly-carboxylate superplasticizer admixture used. The mortar samples were then tested to study the engineering properties, compressive strength and water absorption.

The compressive loading tests were carried out on a compression testing machine of a 500-kN capacity and a loading rate of 0.9 kN/s. The cube specimens measured 50 mm × 50 mm × 50 mm, and the experiment was repeated five times for every mix design before an average was taken for the highest three readings. The test was performed after 3, 7, 14, 28 and 90 days of curing in accordance with ASTM C109M [33]; results are shown in Appendices A–D. The specimens were tested immediately after being taken out of the curing tank.

A water absorption test was conducted when the mortar cubes reached the age of 28 days in accordance with British Standard (BS 1881: Part 122) [34]. Five mortar samples measuring 50 mm × 50 mm × 50 mm were oven-dried at 105 ± 5 °C for 72 ± 2 h undisturbed, but with only free-flowing air to all surfaces of the samples. The specimens were then cooled for 24 ± 0.5 h in a dry airtight vessel. Each specimen was then weighed and immediately immersed in a tank containing clean water maintained at 20 ± 1 °C and with a depth of 25 ± 1 mm over the top of the specimen. The samples were left for 30 ± 0.5 min, wiped dry and weighed again. The absorption rate of these samples was taken as the increase in weight after immersion and presented as the percentage of dry mass.

## 3. Results and Discussion

### 3.1. Slump Flow

**Figure 2:**
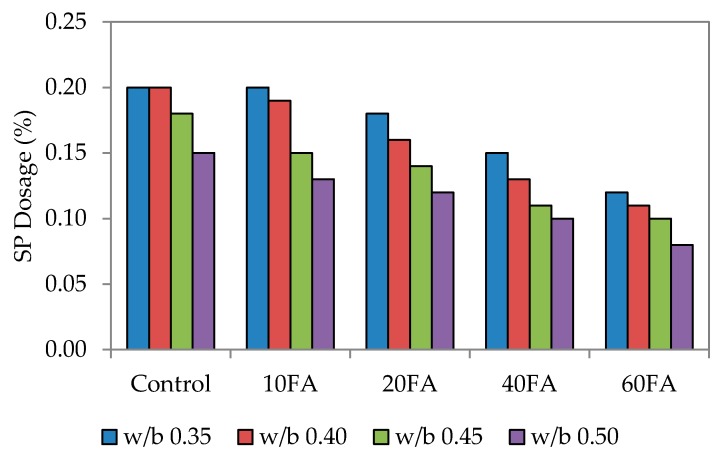
Superplasticizer dosage of normal flowability.

**Figure 3:**
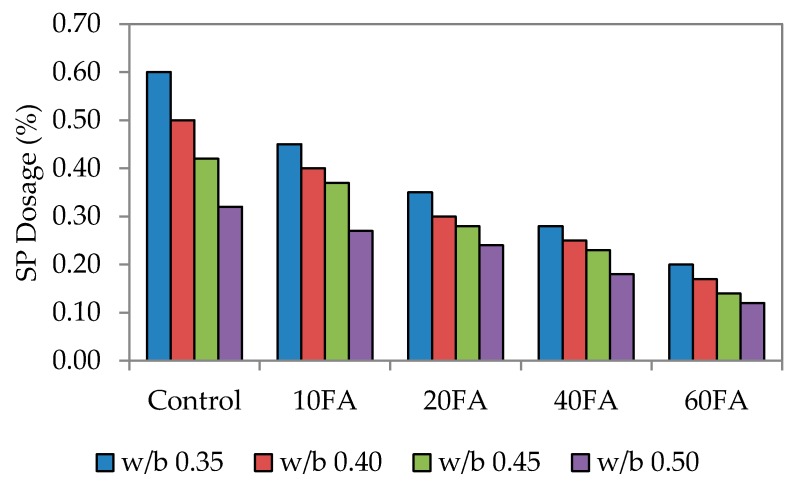
Superplasticizer dosage of high flowability.

**Figure 4:**
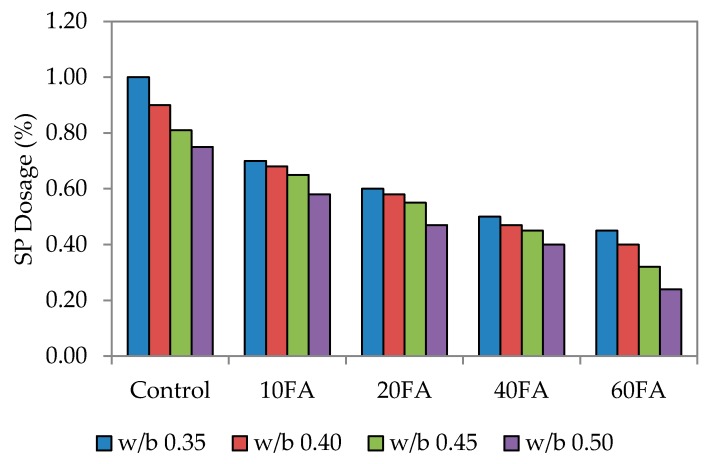
Superplasticizer dosage of self-compacting flowability.

As mentioned earlier, the ability and efficiency of self-compaction is measured through flowability, passing ability and also resistance to segregation. In this study, a mini slump flow test was carried out to check that each particular test mix had achieved the targeted flowability. This range has been previously used by Şahmaran *et al*, Khaleel *et al.* and Turk *et al.* [12,35,36]. Figure 2, Figure 3 and Figure 4 show the dosage of superplasticizer for the targeted slump flow. Generally, the flowability of the mixes had improved with the incorporation of fly ash, possibly due to the round and spherical shape of the particles that had enhanced the rolling capability and the lubrication between particles.

The fineness of powdered materials, such as fly ash, is highly beneficial in improving the workability of mixes and reducing bleeding effects. Fly ash also gives the mixes better cohesiveness and plasticity. Here, the superplasticizer (SP) dosage was the only dependent variable that would affect the targeted slump flow. The conclusion drawn was that the SP dosage utilized is highly dependent on the percentage of fly ash replacement level and workability. By increasing the fly ash replacement level, the workability generally improves, particularly for normal mixes. Figure 2 also shows that increasing the fly ash level has enhanced the workability, although a lower SP dosage would have been sufficient to achieve the targeted slump. A published study has mentioned that the mortar flowing ability decreased with higher SP dosages. The finding of this study is that the flowing ability can be increased with a higher SP dosage, provided that is at a lower water-binder ratio [6].

For control mixes without any fly ash added, the highest SP dosage was recorded at 1.0%. At the maximum fly ash replacement level of 60%, the lowest SP dosages were at 0.14%, 0.28% and 0.56%. This increase in SP dosage is due to the combined effect of greater paste volume and reduced fine material content that has decreased the flowing resistance of the mortar. A similar trend was noticed for water to binder (w/b) ratios of 0.40, 0.45 and 0.50. However, for 60% fly ash replacement, the SP dosage needed to be reduced significantly, and this was the same for every mix irrespective of the w/b ratio. This resulted in lower mortar density since the density of fly ash (2.27) is much lower compared to that of cement [25].

The significant relationship between slump flow and SP dosage is apparent, whereby increasing the SP dosage leads to greater flowability. Resistance of the fresh mortar to flow decreases in this case because of the liquefying action that consequently increases the flow spread of mortar. The overall results have also shown that the w/b ratio plays a vital role in this; an increase in the w/b ratio lowered the paste volume and increased the fine material content to produce a much lower flow spread.

### 3.2. V-Funnel

**Figure 5:**
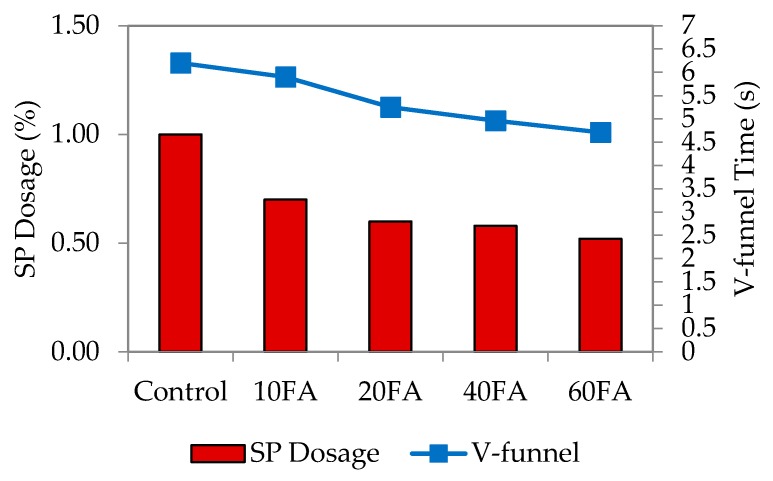
V-funnel time and SP dosage at w/b 0.35.

**Figure 6:**
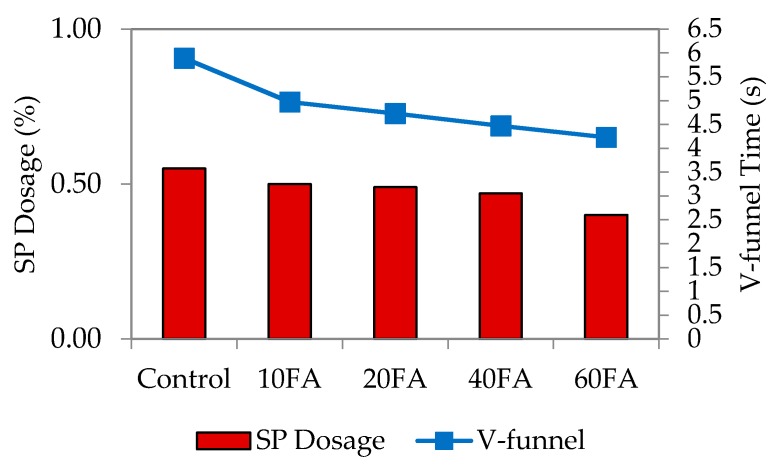
V-funnel time and SP dosage at w/b 0.40.

**Figure 7:**
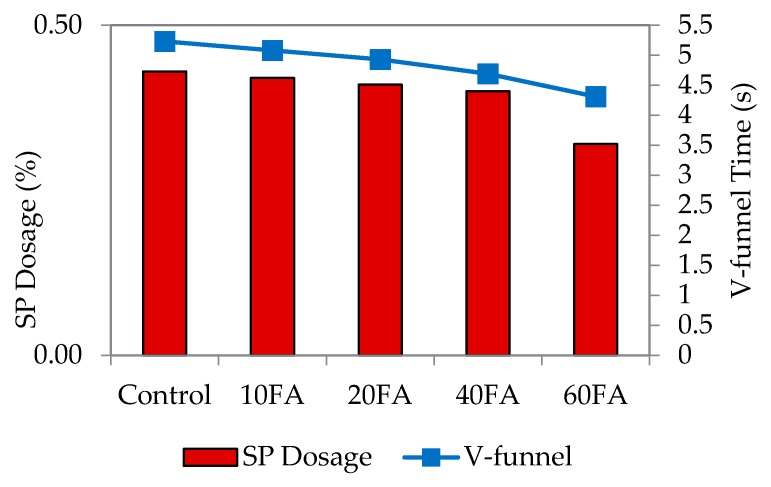
V-funnel time and SP dosage at w/b 0.45.

**Figure 8:**
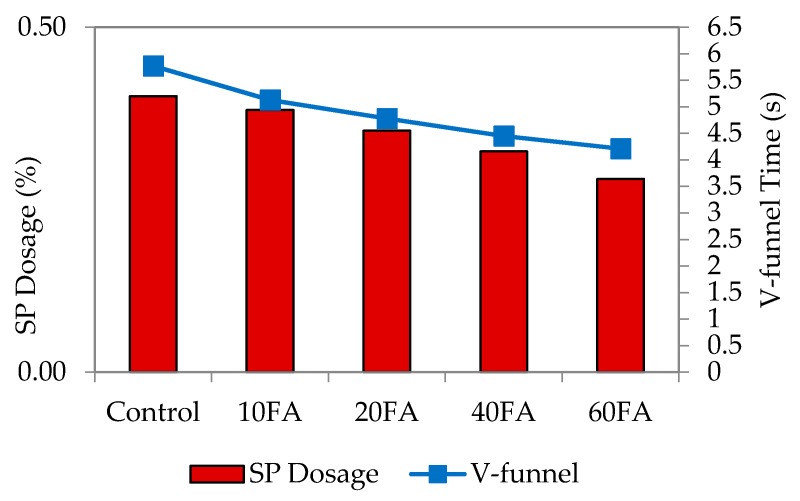
V-funnel time and SP dosage at w/b 0.50.

The V-funnel test assesses the viscosity, as well as the passing and filling ability of self-compacting concrete in particular [3]. It was carried out to ensure that the filling and passing ability is satisfactory. Resistance to segregation was assessed through visual observation during casting to ensure that the mixes are in a homogenous state. Figure 5, Figure 6, Figure 7 and Figure 8 represent the relationship among SP dosage, V-funnel time and replacement levels for w/b 0.35, 0.40, 0.45 and 0.50, with the results showing a similar trend for the reduction in SP dosage and V-funnel time observed. All figures showed that the control specimen needed a high percentage of SP dosage, and this brought higher V-funnel time, to achieve the required flow spread of between 240 and 260 mm for SCM. For every w/b, an increase in replacement level and a decrease in SP dosage shortened the time required for the mixes to completely flow from the mini-V-funnel.

The addition of fly ash at replacement levels of 10%, 20%, 40% and 60% proportionally reduced the V-funnel time. The obvious reason is that the liquefying and dispersing actions of the SP decreased flow resistance. For control mixes at a lower w/b ratio, a large variation between V-funnel time and SP dosage was observed, while it was smaller for that with a higher w/b ratio. The mix proportions indicate that binder proportions are low with less water added into the mixes, which can be attributed to less SP present in the mixes.

**Figure 9:**
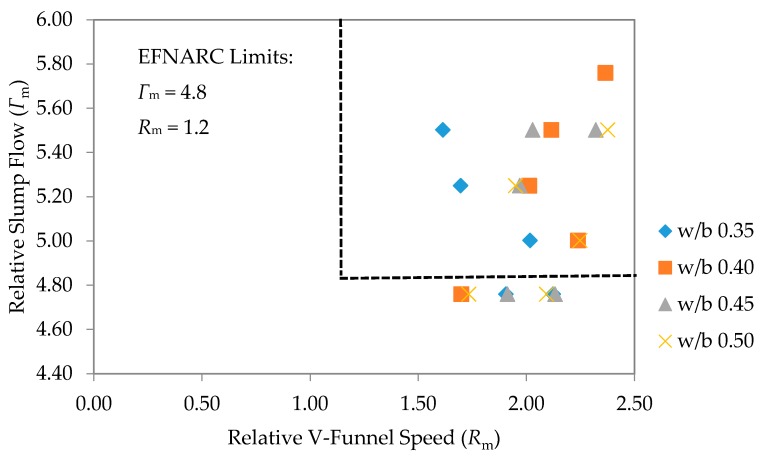
Relative slump flows and relative V-funnel speed.

Figure 9 shows the relationship between the relative slump flow (*Г*_m_) and the relative funnel time speed (*R*_m_). The results obtained are within the limits set by European Federation of National Assoctions Representing for Concrete (EFNARC) [3], which is a relative slump flow, (*Г*_m_) = 4.8, and relative funnel time speed, (*R*_m_) = 1.2. Some data fell within the stipulated boundary belonged to the 60% fly ash replacement at higher w/b ratios of 0.45 and 0.5. Some previous studies had also reported similar trending of relative slump flow to relative V-funnel speed [12].

### 3.3. Environmental Sustainability Performance

The approach by Henry *et al.* [37] in calculating the CO_2_ footprint of concrete when using recycled aggregate has been adopted to understand the environmental sustainability performance of the self-compacting mortar mixes. CO_2_ footprint was calculated based on the SCM mix proportions (kg/m^3^) shown in Table 1 and Table 2 and the CO_2_ emissions of concrete constituent in Malaysia (kg-CO_2_/tonne) shown in Table 4. The product of multiplication was adopted in the calculation of CO_2_ footprints by using data from the mix proportion and CO_2_ emission of concrete constituent [21].

**Figure 10:**
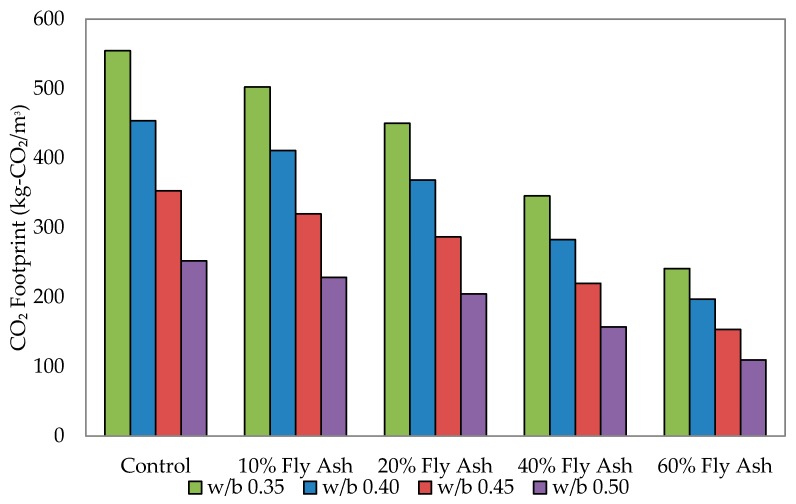
CO_2_ footprint of self-compacting mixes.

The CO_2_ footprint of self-compacting mixes for each w/b ratio is presented in Figure 10. The reduction in CO_2_ footprint was directly proportional to the reduction in the w/b ratio and the amount of fly ash replacement. When comparing the CO_2_ footprint to the control mixes, the benchmark set was that 100% of CO_2_ emission was assumed to have been released from the control mixes (100% of ordinary Portland cement gives 100% of CO_2_ release). Following this trend, at 10% fly ash replacement, it was found that 9.42% lesser CO_2_ had been released, followed by 20% of fly ash replacement at 18.85% and 40% of fly ash replacement at 37.69%. The reduction was highest at 60% of fly ash replacement, where a 56.54% decrease was recorded.

This positive observation shows that, by replacing fly ash to a maximum level of 60%, the CO_2_ footprint can be significantly reduced by more than 50% “to reduce the CO_2_ emission in concrete mixes by minimizing the cement content, replacing it with other minerals” [38]. This will be highly beneficial in promoting environmental and economic sustainability.

Table 5 shows overall comparison of CO_2_ emission from Malaysia, Japan, Australia, United Kingdom and South Africa presented by Putri *et al.* [21], Henry *et al.* [37], Flower *et al.* [22], The Concrete Centre [39] and Theodosiu [40], respectively. In this study, the CO_2_ footprint was calculated based on the SCM mix proportion (kg/m^3^), as shown in Table 1 and Table 2, while CO_2_ emissions of concrete constituent in Malaysia (kg-CO_2_/tonne) were determined as shown in Table 4. The differences in some data produced might be caused by the assumption of energy consumption made for each country with due inference made of the distance for transporting the materials [40].

### 3.4. Compressive Strength versus Environmental Sustainability

**Figure 11:**
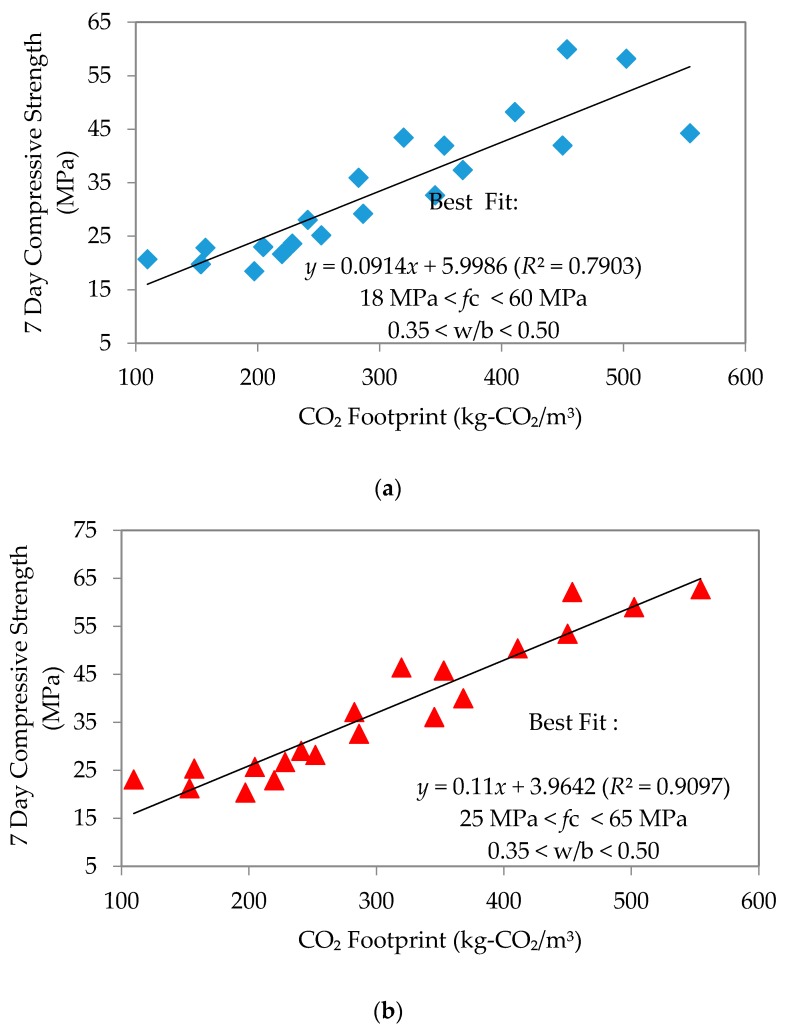

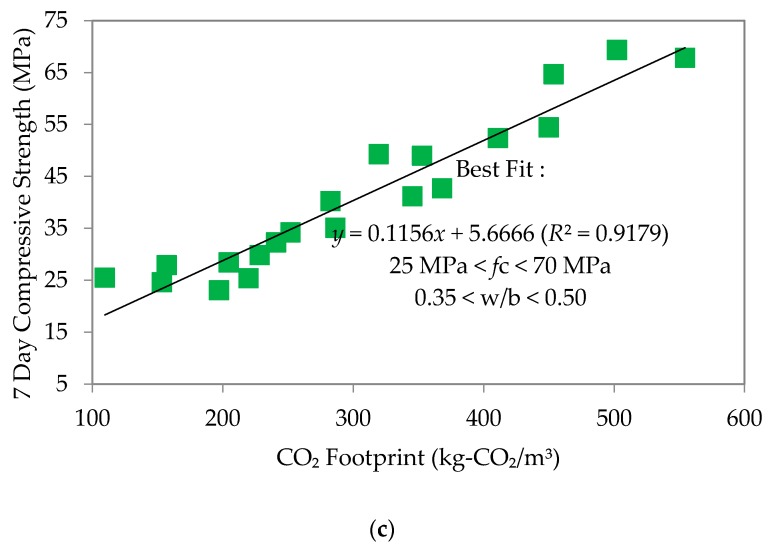
Compressive strength to CO_2_ footprint on seven days’ strength. (**a**) Normal flowability; (**b**) High flowability; (**c**) Self-compacting flowability.

**Figure 12:**
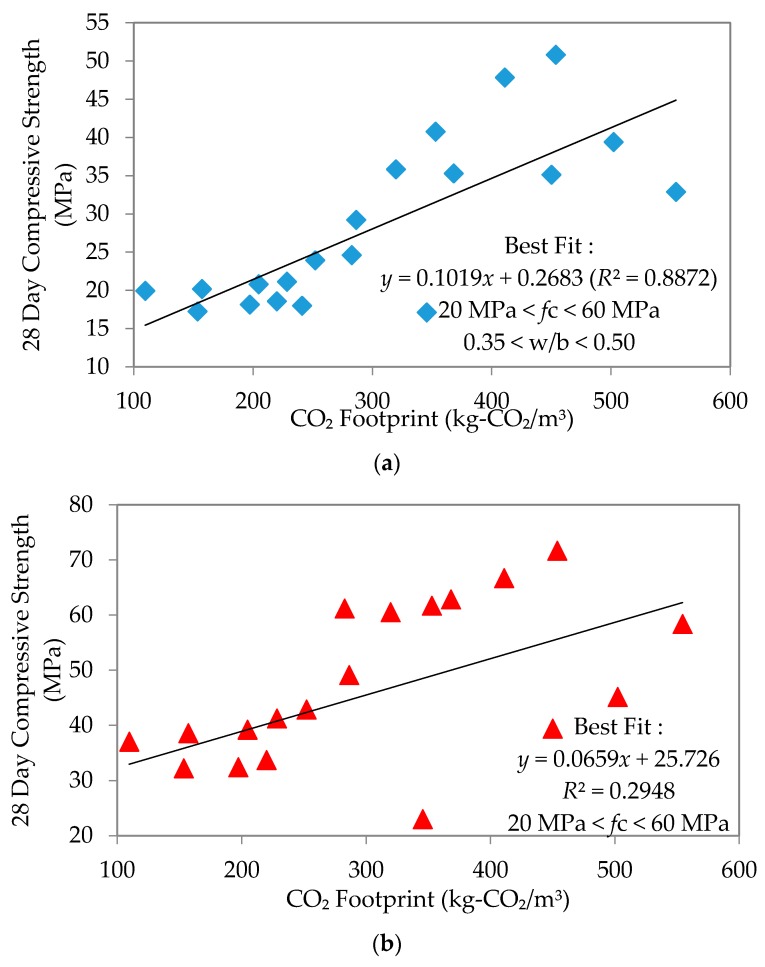

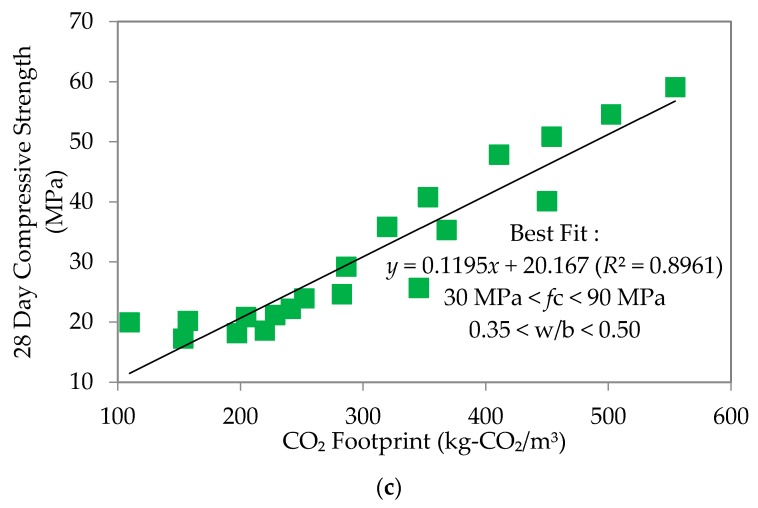
Compressive strength to CO_2_ footprint on 28 days’ strength. (**a**) Normal flowability; (**b**) High flowability; (**c**) Self-compacting flowability.

**Figure 13:**
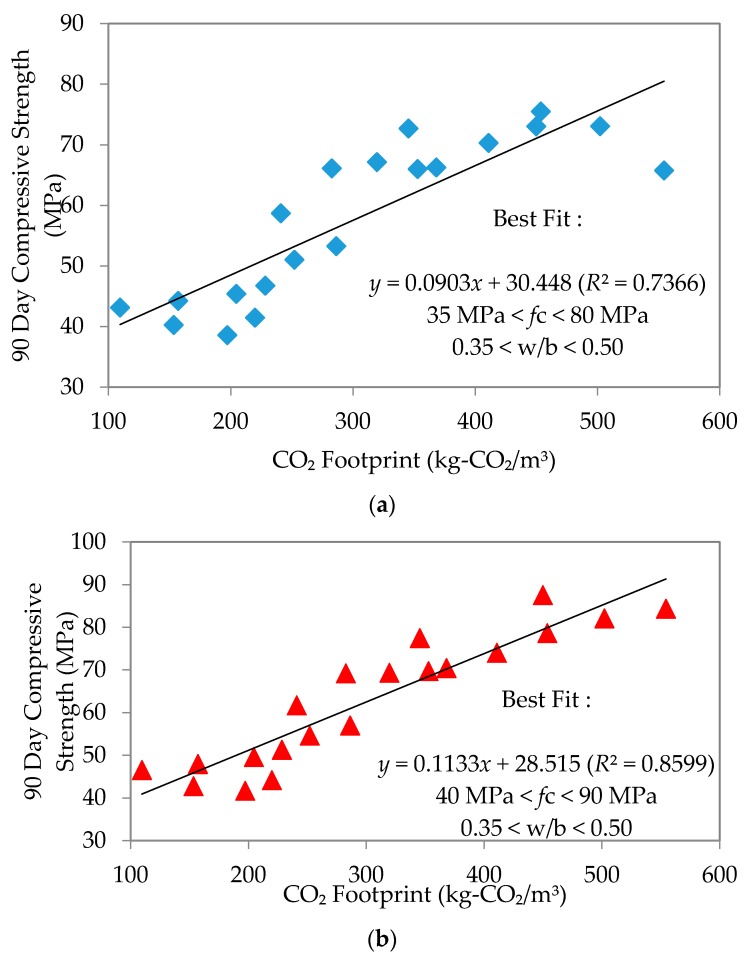

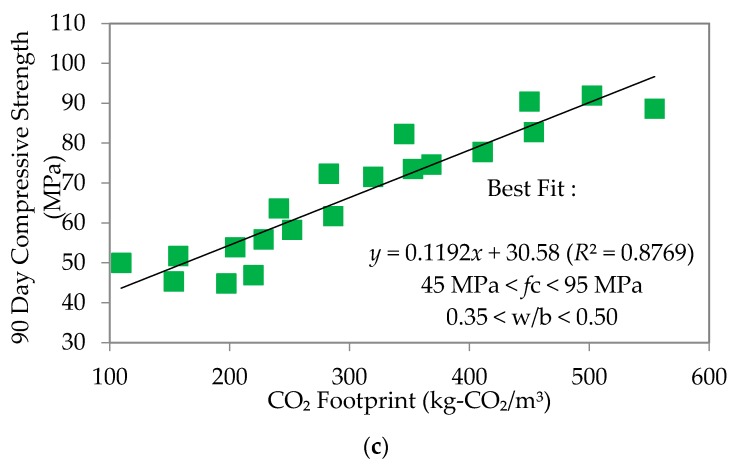
Compressive strength to CO_2_ footprint on 90 days’ strength. (**a**) Normal flowability; (**b**) High flowability; (**c**) Self-compacting flowability.

This section shall discuss the relationship between compressive strength and CO_2_ footprint at 7, 28 and 90 days, interpreted through the plots of “*n*” day compressive strength against CO_2_ footprint. The produced mortar samples were found to have comparable workability after being cured for 7, 28 and 90 days in particular; the linearity of these plots produced the best-fit equations and coefficients of regression as shown in Figure 11, Figure 12 and Figure 13, respectively. Linear equations established for each type of flow are summarized in Table 6 as follows.

The equations clearly showed that an increase in the line gradient has produced a much higher range of compressive strength for all mixes. A lower CO_2_ footprint showed lower strength achieved and *vice versa*. Interestingly, self-compacting flow seems to give better strength, yet with a low CO_2_ footprint. The strength at early age was an average of 39, 45 and 47.5 MPa for normal, high and self-compacting flow, respectively. At 28 days, these became an average of 50, 52.5 and 60 MPa, respectively. This continued to increase such that by the age of 90 days, the average for normal flow, high flow and SCM was 57.5, 65 and 70 MPa, respectively. The observed trend is that SCM had the largest increase in compressive strength through time. Furthermore, the coefficient of regression (*R*^2^) established for 7, 28 and 90 days’ strength to the CO_2_ footprint for each mix depicted a good correlation with an average of 0.873, 0.871 and 0.824, respectively. The linear equation established between 7, 28 and 90 days’ compressive strength and CO_2_ footprint was only applicable for w/b ratios of 0.35–0.50 for all types of flow.

### 3.5. Determination of Optimum Mixes

**Figure 14:**
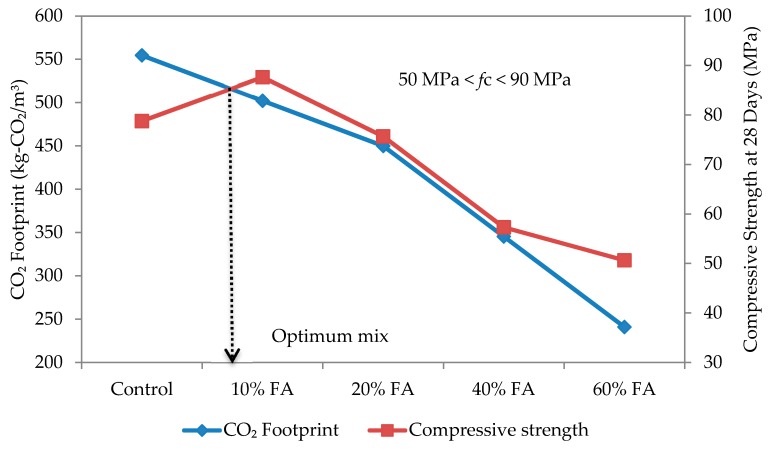
Optimum mix for self-compacting mortar at w/b 0.35.

**Figure 15:**
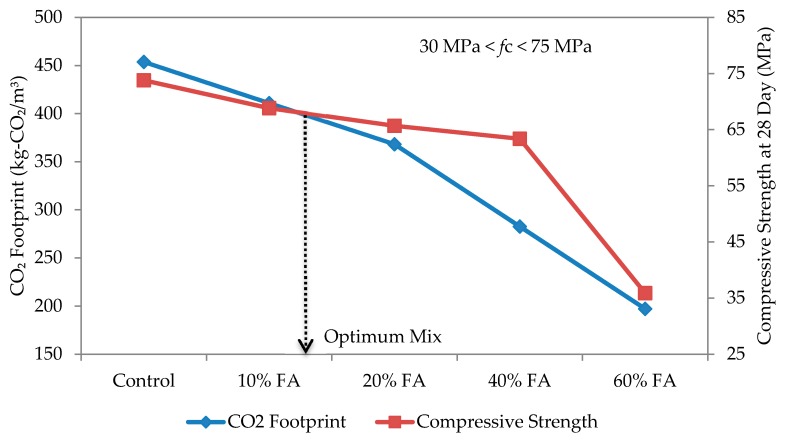
Optimum mix for self-compacting mortar at w/b 0.40.

**Figure 16:**
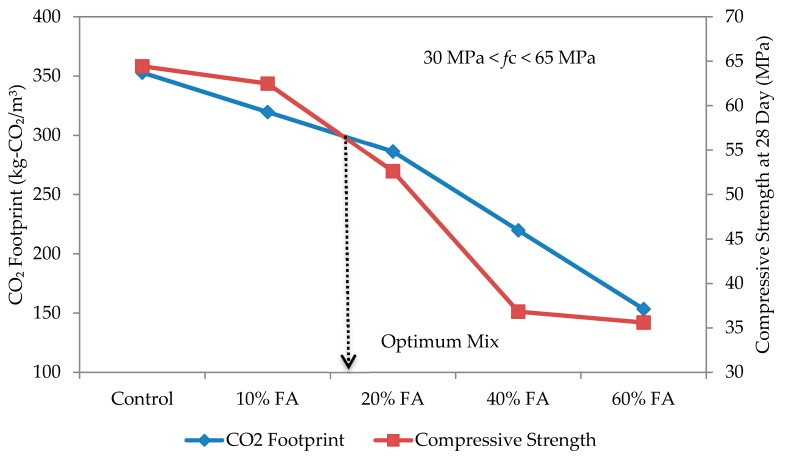
Optimum mix for self-compacting mortar at w/b 0.45.

**Figure 17:**
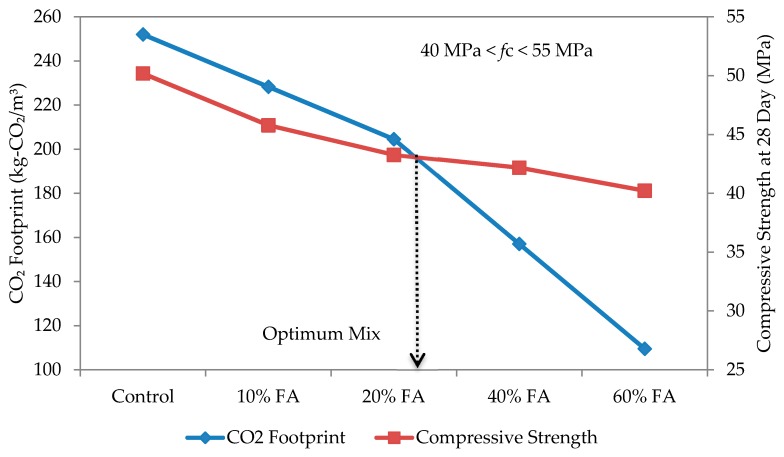
Optimum mix for self-compacting mortar at w/b 0.50.

The optimum mix for self-compacting mortar was determined based on the relationship between compressive strength and CO_2_ footprint, as shown in Figure 14, Figure 15, Figure 16 and Figure 17 for w/b ratios of 0.35, 0.40, 0.45 and 0.50. The intersection point between these two variables was taken as the optimum mix.

Based on Figure 14 for a w/b ratio of 0.35, the optimum mix is when the replacement rate of cement with fly ash is 10%. A similar trend has been observed for all other w/b ratios and over 50% of the specimens, except for the w/b ratio of 0.5, which thus shows that a 10% replacement rate gives the best mix design for SCC. At w/b 0.50, the intersection fell on 20% fly ash replacement. The results suggest that, rather than w/b ratio, the type of fly ash plays a more dominant role in affecting the strength and CO_2_ footprint of the SCM mixes. In this study, a Class F fly ash (refer to Table 3) having low calcium oxide (CaO) and higher silicon dioxide (SiO_2_) was used. Once the compressive strength and CO_2_ footprint surpassed the optimum point, both started to decrease. At this point, the fly ash is only functioning as a filler material. Nevertheless, this also shows that, while the optimum replacement rate is at 10%, it can be increased up to 60% in spite of the recommended 40% or less by Khatib [41] to elevate the utilization rate of fly ash, and a high strength mortar can still be produced. Furthermore, this is highly possible if early strength is not an important factor [42]. In the study of Khatib [41], replacing 40% of ordinary Portland cement with fly ash had resulted in a higher strength of more than 65 N/mm^2^ after 56 days of curing.

### 3.6. Durability versus Environmental Sustainability

Generally, the addition of fly ash decreased the water absorption and elevated the durability performance of the mortar samples. It has been reported that high quality mortar samples generally have a water absorption rate of less than 5% [43,44]. It appears that the water transportation for all mortar samples is affected by two factors—the pores of the cement paste and the interfacial transition zone (ITZ) between the cement paste and the aggregate [31]. It is noted that the volume of water absorption in the mortar specimens corresponds to the degree of porosity, which becomes higher in mortar added with fly ash compared to that without. The proportion of aggregate in the mixture was designed to decrease as the w/b ratio decreases (see Table 1 and Table 2). Therefore, the absolute value of the porosity in the specimens would decrease along with the w/b ratio.

Carbonation is the reaction of carbon dioxide in the environment with calcium hydroxide in cement paste with the presence of water. Since the carbonation reaction of mortar containing fly ash is low, this improves the durability of concrete, where it becomes denser and more resistant in an aggressive environment. Consequently, it reduces the corrosion rate and maintenance costs.

Water absorption is an indicator of durability, where lower water absorption indicates a low w/b ratio and better durability. In this study, the SCM with a high percentage of fly ash replacement gave the highest water absorption. This is attributed to the increase in paste volume due to the lower specific gravity of fly ash, which has enhanced the capillary pore volume and water absorption [45].

**Figure 18:**
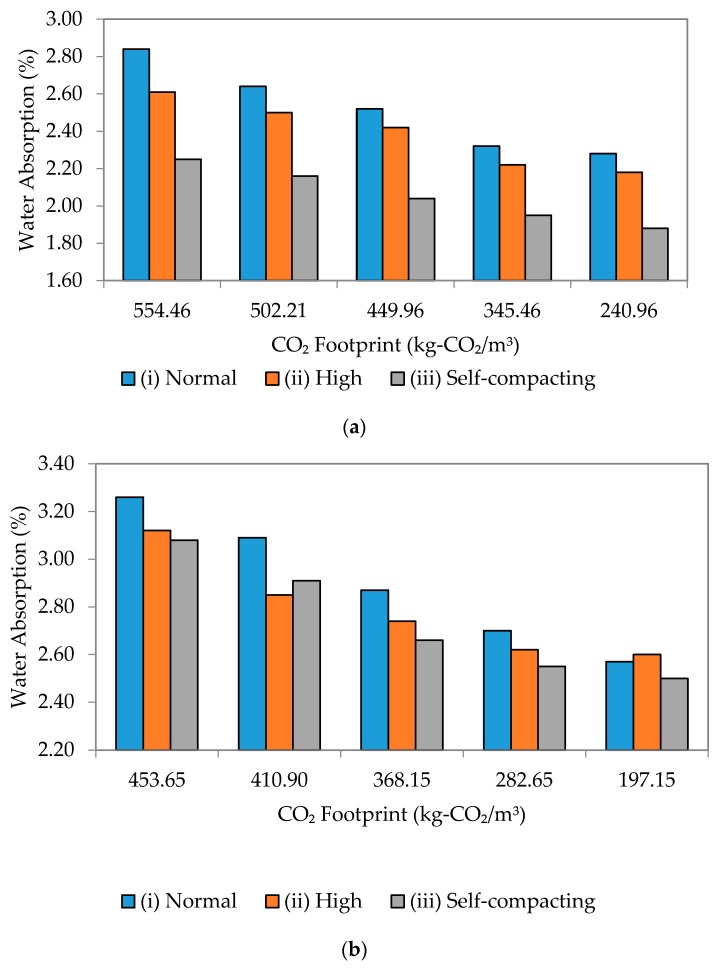
Water absorption to CO_2_ footprint of different flowability. (**a**) w/b 0.35; (**b**) w/b 0.40.

**Figure 19:**
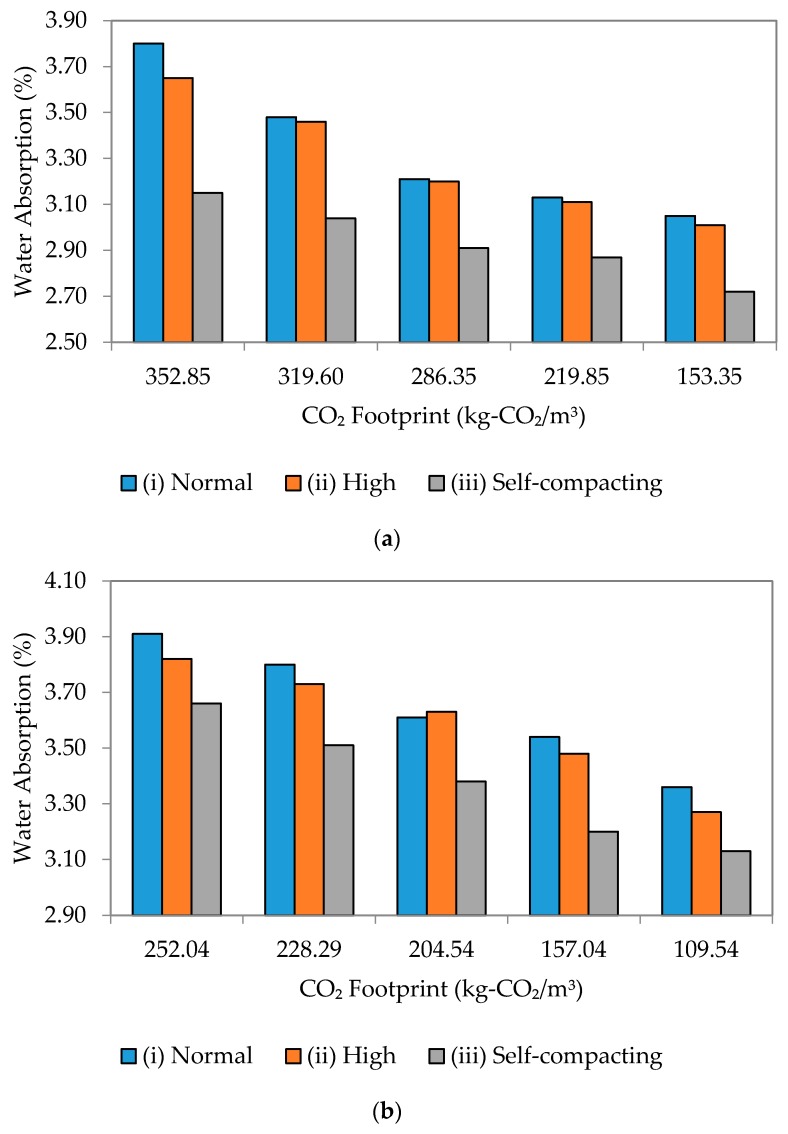
Water absorption to CO_2_ footprint of different flowability. (**a**) w/b 0.45; (**b**) w/b 0.50.

Figure 18 and Figure 19 show the relationship between water absorption and the environmental sustainability impact in terms of CO_2_ footprint for w/b ratios of 0.35, 0.40, 0.45 and 0.50 for all flow mixes. Generally, a positive linear relationship has been established for all cases.

In Figure 18, with the equations gathered, a lower absorption rate has been achieved with a similar CO_2_ footprint value. The observation showed that the dominant factor in determining the relationship between durability and environmental sustainability is highly dependent on the w/b ratio rather than the type of flow. A low w/b ratio seems to provide better performance in terms of durability since it has fewer pores, specifically after adding the mineral admixture. Hence, from the analysis carried out, it can be concluded that SCM gave the lowest water absorption with the lowest CO_2_ footprint.

The relationship between durability and environmental sustainability for w/b ratios of 0.45 and 0.50 produced a very similar trend, whereby a lower slope meant a lower absorption rate and CO_2_ footprint (see Figure 19). All-in-all, there exists a strong correlation specifically for SCM between CO_2_ footprint and water absorption. This also means that the correlation is also strong between the environmental sustainability and durability of concrete; with a low CO_2_ footprint (less environmental impact), the water absorption becomes low, as well (low durability), and *vice versa*.

## 4. Eco-Points of OPC-Fly Ash Self-Compacting Mortar Mixes

The reduction of greenhouse gases is a priority for all industries. Currently, the electrical power industry accounts for approximately 37% of the CO_2_ emissions generated globally [46]. So far, relentless efforts have been poured into developing renewable energy, such as hydro power, solar power and wind power [47]. In this study, the CO_2_ footprint was identified by capturing all constituents formed from cradle to gate production of the SCM, which includes transportation and energy (fuel, diesel and electricity) until the pre-construction stage [22]. The ecological value or eco-point is proposed to describe all associated processes that may incur CO_2_ emission of OPC-fly ash in SCM mixes. These eco-points optimize the utilization of waste materials to mainly reduce Portland cement’s consumption and also to decrease landfill disposal [48], which means the quantifying of CO_2_ emission is mainly associated with the manufacture and placement of cement mortar, as well as the impact of landfill disposal. For now, the environmental impacts identified include consumption of non-renewable raw materials, energy sources and destruction of biodiversity. Not only that, since the cost of aggregates is very dependent on the transport distances, leading to extraction operations near construction sites, this multiplies the number of quarries and their biodiversity impacts. The evaluation of eco-points as suggested by Yang *et al.* [23] is described as follows:

Eco-points = CO_2_ footprint + Transportation + Energy (Diesel + Electricity) (1)

**Figure 20:**
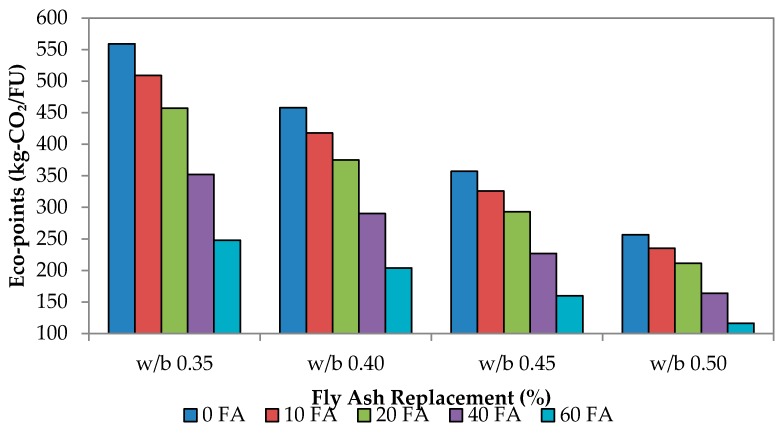
Eco-points of self-compacting mixes.

Eco-points of w/b 0.35 for self-compacting mixes are tabulated in Table 7, Table 8, Table 9, Table 10 and Table 11. Table 7, Table 8, Table 9, Table 10 and Table 11 present the eco-point assessment for SCM from 0% up to 60% fly ash replacement. This assessment measures the carbon emission of three stages: the material and production of 1 m^3^ of mortar; the distance required to transport the mortar; and the energy consumed (diesel oil and electricity) during production, transporting and batching the mixes. The sum of this is termed the “eco-point”. Those for w/b 0.40, 0.45 and 0.50 are summarized graphically in Figure 20. Fundamentally, the trending has been the same for all w/b ratios where more energy is required when no fly ash is added, which in turn generated more eco-points. With this, it also indicates that, the higher the percentage of fly ash, the lesser the energy produced, because the fly ash particles have filled up the spaces and voids. In SCM, this is even more effortless, since no vibration is needed, thus requiring even lesser energy. Consequently, it decreases the eco-points and further lessens the CO_2_ footprint. Results showed that total production emission reduced by 56% from 558.87 kg-CO_2_/FU down to 247.76 kg-CO_2_/FU when fly ash replacement rate increased from 0%–60% (maximum). This is translated to a reduction of 80% in eco-points (assuming that the energy consumption rate of production with 0% fly ash is at 100%). Therefore, this analysis has shown how the eco-points of any particular mix associated with fly ash replacement can achieve considerable savings associated with CO_2_ emission. 

### 4.1. Fly Ash Landfill

One of the major problems of all coal combustion power plants is the unused fly ash and bottom ash that bring environmental problems, such as air pollution and groundwater contamination, due to the leaching of metals from the ashes, especially the accumulation of the very fine particles of fly ash. The utilization of fly ash in replacing the cement in concrete mixture decreases both energy and CO_2_ emitted during production [49]. In Malaysia, fly ash is an industrial waste material commonly deposited in landfills. This brings no benefits, but rather environmental nuisance [50]. Currently, 3.4–4 billion tonnes of municipal and industrial waste are generated annually, of which non-hazardous industrial waste accounts for 1.2 billion tonnes [51] and the amount of wastes dumped at landfills is radically increasing due to industrial development and urbanization. The production of fly ash in Malaysia is believed to be approaching two million tons annually [52]. Near zero utilization of such waste material occupies land and brings other problems, such as leaching of traceable and hazardous elements [53], as well as contamination of groundwater.

Due to its potential toxic elements (PTEs) production e.g., Cadmium (Cd), Chromium (Cr), Nickel (Ni), Lead (Pb) and organic compound e.g., polychlorinated biphenyls (PCBs), polycyclic aromatic hydrocarbons (PAHs) content, coal fly ash is classified as a scheduled and hazardous solid waste. Uncontrolled land disposal of coal fly ash is likely to cause unnecessary transformation in soil conditions, including contaminating the soil with PTEs, PAHs and PCBs. However, coal fly ash is also a valuable resource of important plant nutrients, e.g., Calcium (Ca), Magnesium (Mg), Pottasium (K), Phosphorus (P), Sulphur (S), Boron (B), Iron (Fe), Copper (Cu) and Zinc (Zn) [54], where the addition of 2%–5% of fly ash to calcareous soils has resulted in better plant growth compared to normal soils [55]. However, when the application exceeds 5%, the crop growth was significantly reduced [54]. Coal residues contain hazardous substances, which also pollute the environment if handled and disposed incorrectly.

The overall assessment was made by setting up distinct boundaries with due reference to the current practices in the industry. The fly ash was used directly from the power plant after the incineration process without any further processing stages. Considering the fact that fly ash is mostly exported to the cement producer before being dumped in the landfill, the boundaries for the assessment of fly ash landfill were limited to a smaller scale analysis. In addition, the fly ash used in the laboratory was obtained as a completely burned final by-product that could be used directly for concrete production without any further processing stages. Hence, it may positively have less impact on energy utilization.

**Figure 21:**
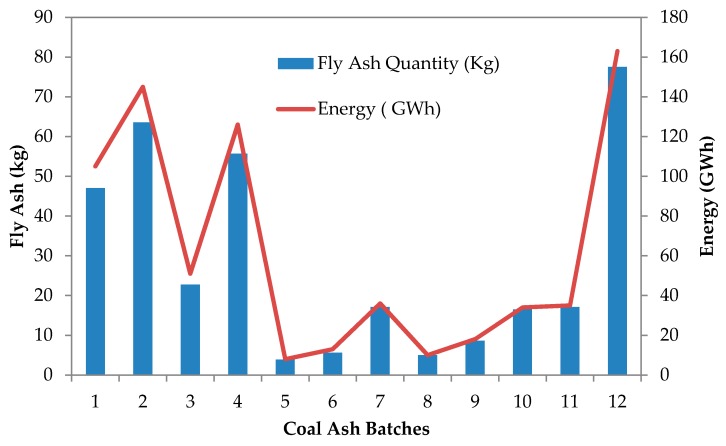
Fly ash data and energy production of a power plant from northern peninsular Malaysia.

**Figure 22:**
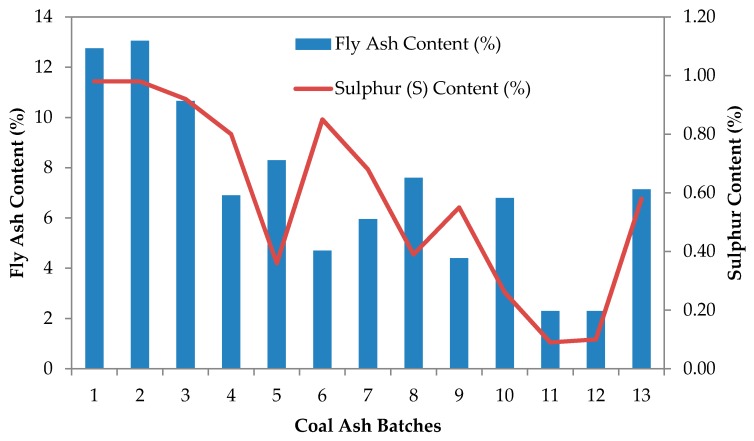
Fly ash–sulfur content of of a power plant from northern peninsular Malaysia.

A power plant in northern part of peninsular Malaysia produces ash at an average of 340,576 tonnes per month [56], and the production breakdown is as illustrated in Figure 21. Generally, approximately 70% of it is sold to cement manufacturers and producers, while the remaining 30% is dumped to the ash pond due to quality control procedures [56]. Figure 22 shows the fly ash–sulfur content of ash produced from the power plant. Based on the data production, 1% fly ash has 0.08% of sulfur in the landfill itself [56]. This also means that, by reducing fly ash disposal at the landfill pond, a further 5%–27% reduction of sulfur content is possible. In addition, this also reduces the disposal area from 15%–91% on average per month (assuming 10%–60% of fly ash utilization in concrete or mortar mixes per month). However, whether or not the reduction in toxicity and occupied area has a significant impact on the environment and ecology has to be further investigated to cover a larger scale.

### 4.2. Fly Ash Contaminant Landfill Savings

**Figure 23:**
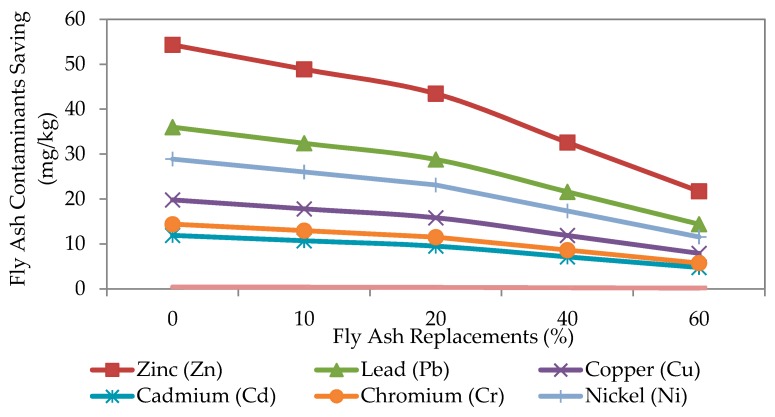
Fly ash contaminant savings.

Fly ash is primarily made up of silica, alumina and iron oxide and is no foreign in concrete and mortar mixes. Fly ash is produced from the combustion of coal production and is normally dumped in landfill ponds. The problem is that it may cause leaching of trace elements, such as zinc, lead, copper, chromium, cadmium, nickel and also mercury into the soil. Nevertheless, blending fly ash with lime can avoid water pollution if the fly ash is used as back-filling material under acid mine drainage conditions. The trace elements’ concentrations are higher in acidic fly ash than alkaline fly ash. Table 12 summarizes several studies conducted by Bie *et al.* [57], Tsiridis *et al.* [58], Jala *et al.* [59], Tripathi *et al.* [60], Pandey *et al.* [61], Riehl *et al.* [62] and Pourrut *et al.* [63] in analyzing trace element in fly ash landfill soil. The standard measurement of contaminated soil reported by the Department of Environmental Malaysia [64] is also included in the analysis. Figure 23 shows the breakdown analysis of fly ash toxicity reduction in contaminated landfill soils. The trend is similar for 10%, 20%, 40% and 60% of fly ash replacement for all trace elements. The high level of zinc (Zn) may also be coming from the plants and due to soil weathering. Mercury (Hg) impact is negligible since the reduction in contamination is similar for 0%–60% fly ash replacement. From the analysis, it is clear that reducing the disposal of fly ash is highly beneficial to the environment.

## 5. Conclusions

The CO_2_ footprint was correlated with the engineering properties of mortar for three different spread flows (normal, high and self-compacting). The experimental results clearly demonstrated that replacing cement with other materials can reduce the environmental impact due to the lower CO_2_ footprint. From the present experimental investigation and assessment of the environmental impact, the following conclusions can be drawn:

1. Considering the strength of mortar and environmental sustainability, the optimum mix for self-compacting mortar is at a 10% replacement rate of cement with fly ash for all mixes.
2. From the plots of CO_2_ footprint against compressive strength at various ages, a 60% replacement of cement with fly ash had decreased more than 50% of the CO_2_ footprint compared to other mixes.
3. The analysis of 28 days’ compressive strength to CO_2_ footprint showed that, with the increase in strength, a linear relationship had been obtained for both variables.
4. The analysis of environmental impact concluded that self-compacting flow gave the lowest water absorption and the lowest CO_2_ footprint.
5. Eco-points of 10% replacement of fly ash were 10.27% higher than 20% fly ash replacement, whilst 22.9% higher than 40% replacement of fly ash.
6. The utilization of fly ash in concrete or mortar can reduce sulfur content in the soil by 5%–27%.
7. The occupied landfill area of fly ash can be reduced by 15%–91% on average.

## Figures and Tables

**Table 1:** Mix proportion w/b 0.35 and 0.40.

Mixture	Water: Binder (w/b)	Water (kg/m^3^)	Cement (kg/m^3^)	Sand (kg/m^3^)	Fly Ash (kg/m^3^)	SP (kg/m^3^)	Superplasticizer (SP) Dosage (%)		
							Normal	High	Self-Compacting
Mix 1A-C	0.35	192.5	550	1100	0	1.10	0.20	-	-
Mix 1B-C		192.5	550	1100	0	3.30	-	0.60	-
Mix 1C-C		192.5	550	1100	0	5.50	-	-	1.00
Mix 2A-10%FA	0.35	192.5	495	1100	55	0.99	0.20	-	-
Mix 2B-10%FA		192.5	495	1100	55	2.23	-	0.45	-
Mix 2C-10% FA		192.5	495	1100	55	3.47	-	-	0.70
Mix 3A-20% FA	0.35	192.5	440	1100	110	0.79	0.18	-	-
Mix 3B-20% FA		192.5	440	1100	110	1.54	-	0.35	-
Mix 3C-20%FA		192.5	440	1100	110	2.64	-	-	0.60
Mix 4A-40%FA	0.35	192.5	330	1100	220	0.53	0.16	-	
Mix 4B-40%FA		192.5	330	1100	220	0.99	-	0.30	-
Mix 4C-40%FA		192.5	330	1100	220	1.91	-	-	0.58
Mix5A-60%FA	0.35	192.5	220	1100	330	0.31	0.14	-	-
Mix 5B-60%FA		192.5	220	1100	330	0.62	-	0.28	-
Mix 5C-60%FA		192.5	220	1100	330	1.14	-	-	0.52
Mix 6A-C	0.40	180	450	900	0	0.90	0.20	-	-
Mix 6B-C		180	450	900	0	1.35	-	0.30	-
Mix 6C-C		180	450	900	0	2.48	-	-	0.55
Mix 7A-10%FA	0.40	180	405	900	45	0.73	0.18	-	-
Mix 7B-10%FA		180	405	900	45	1.13	-	0.28	-
Mix 7C-10% FA		180	405	900	45	2.03	-	-	0.5
Mix 8A-20% FA	0.40	180	360	900	90	0.61	0.17	-	-
Mix 8B-20% FA		180	360	900	90	0.97	-	0.27	-
Mix 8C-20%FA		180	360	900	90	1.76	-	-	0.49
Mix 9A-40%FA	0.40	180	270	900	180	0.43	0.16	-	-
Mix 9B-40%FA		180	270	900	180	0.68	-	0.25	-
Mix 9C-40%FA		180	270	900	180	1.27	-	-	0.47
Mix 10A-60%FA	0.40	180	180	900	270	0.23	0.13	-	-
Mix 10B-60%FA		180	180	900	270	0.31	-	0.17	-
Mix 10C-60%FA		180	180	900	270	0.72	-	-	0.40

**Table 2:** Mix proportion w/b 0.45 and 0.50.

Mixture	Water: Binder (w/b)	Water (kg/m^3^)	Cement (kg/m^3^)	Sand (kg/m^3^)	Fly Ash (kg/m^3^)	SP (kg/m^3^)	Superplasticizer(SP) Dosage (%)		
							Normal	High	Self-Compacting
Mix 11A-C	0.45	157.5	350	700	0	0.63	0.18	-	-
Mix 11B-C		157.5	350	700	0	1.09	-	0.31	-
Mix 11C-C		157.5	350	700	0	1.51	-	-	0.43
Mix 12A-10%FA	0.45	157.5	315	700	35	0.47	0.15	-	-
Mix 12B-10%FA		157.5	315	700	35	0.95	-	0.30	-
Mix 12C-10% FA		157.5	315	700	35	1.32	-	-	0.42
Mix 13A-20% FA	0.45	157.5	280	700	70	0.39	0.14	-	-
Mix 13B-20% FA		157.5	280	700	70	0.56	-	0.20	-
Mix 13C-20%FA		157.5	280	700	70	1.06	-	-	0.38
Mix 14A-40%FA	0.45	157.5	210	700	140	0.27	0.13	-	-
Mix 14B-40%FA		157.5	210	700	140	0.32	-	0.15	-
Mix 14C-40%FA		157.5	210	700	140	0.67	-	-	0.32
Mix 15A-60%FA	0.45	157.5	140	700	210	0.15	0.11	-	-
Mix 15B-60%FA		157.5	140	700	210	0.34	-	0.24	-
Mix 15C-60%FA		157.5	140	700	210	0.28	-	-	0.20
Mix 16A-C	0.50	125	250	500	0	0.38	0.15	-	-
Mix 16B-C		125	250	500	0	0.75	-	0.30	-
Mix 16C-C		125	250	500	0	1.00	-	-	0.4
Mix 17A-10%FA	0.50	125	225	500	25	0.29	0.13	-	-
Mix 17B-10%FA		125	225	500	25	0.61	-	0.27	-
Mix 17C-10% FA		125	225	500	25	0.86	-	-	0.38
Mix 18A-20% FA	0.50	125	200	500	50	0.24	0.12	-	-
Mix 18B-20% FA		125	200	500	50	0.48	-	0.24	-
Mix 18C-20%FA		125	200	500	50	0.70	-	-	0.35
Mix 19A-40%FA	0.50	125	150	500	100	0.15	0.10	-	-
Mix 19B-40%FA		125	150	500	100	0.27	-	0.18	-
Mix 19C-40%FA		125	150	500	100	0.48	-	-	0.32
Mix 20A-60%FA	0.50	125	100	500	150	0.08	0.08	-	-
Mix 20B-60%FA		125	100	500	150	0.15	-	0.15	-
Mix 20C-60%FA		125	100	500	150	0.28	-	-	0.28

**Table 3:** Chemical composition and physical properties.

Component (%)	SiO_2_	Al_2_O_3_	CaO	Fe_2_O_3_	MgO	SO_3_	K_2_O	TiO_2_	CO_2_	Specific Gravity	Fineness (m^2^/kg)
Cement	18.47	4.27	64.09	2.06	2.08	4.25	0.28	0.11	4.2	3.11	390
Fly Ash	48.2	30.7	8.31	-	-	0.78	1.06	-	-	2.27	469

**Table 4:** CO_2_ emissions of concrete constituent in Malaysia [21].

Element	Specific Emission (kg-CO_2_/tonne)
Aggregate	4
Cement CEM I	1000
Fly Ash	50
Water	0.3
Admixtures	0.2

**Table 5:** Overall CO_2_ emission comparison.

Concrete Materials	Putri *et al.* [21]	Henry *et al.* [37]	Flower *et al.* [22]	The Concrete Centre [39]	Theodosiu [40]
Normal Portland cement	1000	767	822	819	818
Aggregate	4	3	32	4	5
Fly Ash	50	20	27	4	2
GGBS	*	27	143	52	128
Admixture	0.2	*	*	380	220
Water	0.3	*	*	*	*
Unit	Kg-CO_2_/tonne		Kg-CO_2_-e/tonne		

GGBS and * are the notations for ground granulated blast slag and “not mentioned”, respectively.

**Table 6:** Linear equations for compressive strength at 7, 28 and 90 days’ strength.

Tested Age	Normal Mixes	High Mixes	Self-Compacting Mixes
7 Days	*Y* = 0.0914*x* + 5.9986 (*R*^2^ = 0.7903) 18 MPa < *f*c < 60 Mpa	*Y* = 0.11*x* + 3.9642 (*R*^2^ = 0.9097) 25 MPa < *f*c < 65 Mpa	*Y* = 0.1156*x* + 5.666 (*R*^2^ = 0.9179) 25 MPa < *f*c < 70 Mpa
28 Days	*Y* = 0.1005 + 17.525 (*R*^2^ = 0.8348) 25 MPa < *f*c < 75 Mpa	*Y* = 0.1134*x* + 18.1666 (*R*^2^ = 0.8816) 30 MPa < *f*c < 75 Mpa	*Y* = 0.1195*x* + 20.167 (*R*^2^ = 0.8961) 30 MPa < *f*c < 90 Mpa
90 Days	*Y* = 0.0903*x* + 30.448 (*R*^2^ = 0.7366) 35 MPa < *f*c < 80 Mpa	*Y* = 0.1133*x* + 28.515 (*R*^2^ = 0.8599) 40 MPa < *f*c < 90 Mpa	*Y* = 0.1192*x* + 30.580 (*R*^2^ = 0.8769) 45 MPa < *f*c < 95 Mpa

Where: *Y* is the compressive strength, while *X* is the CO_2_ footprint; *f*c denotes the range of flexural compression.

**Table 7:** Eco-points’ assessment for OPC + 0% fly ash compacting mortar (control).

Functional Unit (FU): m^3^	Material and Production				Transportation	Energy	Total Emission
Materials	A (Mix Proportion)	B ^1^ (CO_2_ Emission)	A.B (CO_2_ Footprint)	D ^2^	E ^2^	F ^3^	(A.B).(D.E.F)
Unit	(kg/m^3^)	(kg-CO_2_/m^3^)	(kg-CO_2_)	km	(CO_2_-kg/kg-km)	(CO_2_/FU)	(kg-CO_2_/FU)
OPC	550	1000	550	100	5.18 × 10⁻⁵	4.392	12.51
Fly Ash	0	50	0	200	5.18 × 10⁻⁵	**-	**-
Fine Aggregate	1100	4	4.4	50	5.18 × 10⁻⁵	**-	**-
Water	192.5	0.3	0.05775	-	6.30 × 10⁻⁵	**-	**-
Admixture	1	0.2	0.0002	50	2.21 × 10⁻⁴	**-	**-
Total Production	1843.5		554.458	400	4.394 × 10⁻⁵	4.392	558.87

^1,2,3^ Data from Putri *et al.* [21], Yang *et al.* [23] and Theodosiu [40], respectively; “.” Denotes function of multiplications; **- not captured, since control is with 0% fly ash replacement.

**Table 8:** Eco-points’ assessment for OPC + 10% fly ash compacting mortar.

Functional Unit (FU): m^3^	Material and Production				Transportation	Energy	Total Emission
Materials	A (Mix Proportion)	B ^1^ (CO_2_ Emission)	A.B (CO_2_ Footprint)	D ^2^	E ^2^	F ^3^	(A.B).(D.E.F)
Unit	(kg/m^3^)	(kg-CO_2_/m^3^)	(kg-CO_2_)	km	(CO_2_-kg/kg-km)	(CO_2_/FU)	(kg-CO_2_/FU)
OPC	495	1000	495	100	5.18 × 10⁻⁵	4.392	11.26
Fly Ash	55	50	2.75	200	5.18 × 10⁻⁵	2.196	0.0626
Fine Aggregate	1100	4	4.4	50	5.18 × 10⁻⁵	0.17568	0.0020
Water	192.5	0.3	0.05775	-	6.30 × 10⁻⁵	0.013176	*-
Admixture	0.7	0.2	0.00014	50	2.21 × 10⁻⁴	0.008784	*-
Total Production	1843.2		502.208	400	4.394 × 10⁻⁵	6.78564	509.01

*- negligible, since energy produces for water and admixture too small.

**Table 9:** Eco-points’ assessment for OPC + 20% fly ash compacting mortar.

Functional Unit (FU): m^3^	Material and Production				Transportation	Energy	Total Emission
Materials	A (Mix Proportion)	B ^1^ (CO_2_ Emission)	A.B (CO_2_ Footprint)	D ^2^	E ^2^	F ^3^	(A.B).D.E.F
Unit	(kg/m^3^)	(kg-CO_2_/m^3^)	(kg-CO_2_)	km	(CO_2_-kg/kg-km)	(CO_2_/FU)	(kg-CO_2_/FU)
OPC	440	1000	440	100	5.18 × 10⁻⁵	4.392	10.01
Fly Ash	110	50	5.5	200	5.18 × 10⁻⁵	2.196	0.1251
Fine Aggregate	1100	4	4.4	50	5.18 × 10⁻⁵	0.17568	0.0020
Water	192.5	0.3	0.05775	-	6.30 × 10⁻⁵	0.013176	*-
Admixture	0.6	0.2	0.00012	50	2.21 × 10⁻⁴	0.008784	*-
Production	1843.1	1054.5	449.958	400	4.394 × 10⁻⁵	6.78564	456.76

**Table 10:** Eco-points’ assessment for OPC + 40% fly ash compacting mortar.

Functional Unit (FU): m^3^	Material and Production				Transportation	Energy	Total Emission
Materials	A (Mix Proportion)	B ^1^ (CO_2_ Emission)	A.B (CO_2_ Footprint)	D ^2^	E ^2^	F ^3^	(A.B).D.E.F
Unit	(kg/m^3^)	(kg-CO_2_/m^3^)	(kg-CO_2_)	km	(CO_2_-kg/kg-km)	(CO_2_/FU)	(kg-CO_2_/FU)
OPC	330	1000	330	100	5.18 × 10⁻⁵	4.392	7.51
Fly Ash	220	50	11	200	5.18 × 10⁻⁵	2.196	0.2503
Fine Aggregate	1100	4	4.4	50	5.18 × 10⁻⁵	0.17568	0.0020
Water	192.5	0.3	0.05775	-	6.30 × 10⁻⁵	0.013176	*-
Admixture	0.58	0.2	0.000116	50	2.21 × 10⁻⁴	0.008784	*-
Production	1843.08	-	345.458	400	4.394 × 10⁻⁵	6.78564	352.26

**Table 11:** Eco-points’ assessment for OPC + 60% fly ash compacting mortar.

Functional Unit (FU): m^3^	Material and Production				Transportation	Energy	Total Emission
Materials	A (Mix Proportion)	B ^1^ (CO_2_ Emission)	A.B (CO_2_ Footprint)	D ^2^	E ^2^	F ^3^	(A.B).D.E.F
Unit	(kg/m^3^)	(kg-CO_2_/m^3^)	(kg-CO_2_)	km	(CO_2_-kg/kg-km)	(CO_2_/FU)	(kg-CO_2_/FU)
OPC	220	1000	220	100	5.18 × 10⁻⁵	4.392	5.01
Fly Ash	330	50	16.5	200	5.18 × 10⁻⁵	2.196	0.3754
Fine Aggregate	1100	4	4.4	50	5.18 × 10⁻⁵	0.17568	0.0020
Water	192.5	0.3	0.05775	-	6.30 × 10⁻⁵	0.013176	*-
Admixture	0.52	0.2	0.000104	50	2.21 × 10⁻⁴	0.008784	*-
Production	1843.02	-	240.958	400	4.394 × 10⁻⁵	6.78564	247.76

**Table 12:** Fly ash contaminants in soil.

Heavy Metals	Standard Measurement of Contaminated Land (Max) [64]	Standard Measurement of Contaminated Land (Min) [64]	Bie *et al.* [57]	Tsiridis *et al.* [58]	Jala *et al.* [59]	Tripathi *et al.* [60]	Pandey *et al.* [61]	Riehl *et al.* [62]	Lopareva *et al.* [63]
Zinc (Zn)	54.3	6.9	29.32	0.45	79	57.7	82.3	167	85
Lead (Pb)	36	0.18	9.45	0.05	35	20	40.2	97	39
Copper (Cu)	19.8	4	5.23	0.23	0.002	65.8	58.4	57	38
Cadmium (Cd)	11.9	0.09	0.31	0.005	1.9	13.4	42.5	0.03	-
Chromium (Cr)	14.4	0.02	1.05	4.92	330	38.2	40.3	148	46
Nickel (Ni)	28.9	0.7	0.6	0.001	13	44.2	204.8	88	48
Mercury (Hg)	0.42	0.02	0.33	-	-	-	-	-	0.4

## Appendix

**Table A1:** Compressive strength for w/b 0.35.

Mixture	Compressive Strength (MPa) *				
	3 Days	7 Days	14 Days	28 Days	90 Days
C-0.35-N	32.89 *1.20*	44.25 *1.36*	55.70 *0.28*	62.95 *0.30*	65.74 *0.13*
C-0.35-H	58.39 *0.52*	62.76 *0.19*	70.05 *0.41*	74.61 *0.23*	84.38 *1.94*
C-0.35-SCM	59.06 *0.65*	67.81 *0.85*	74.71 *1.34*	78.77 *0.72*	88.62 *0.38*
10FA-0.35-N	39.41 *0.58*	58.20 *0.19*	65.37 *1.94*	70.13 *1.89*	73.05 *1.89*
10FA-0.35-H	45.16 *0.64*	59.03 *0.48*	67.71 *0.45*	77.88 *0.16*	82.10 *0.55*
10FA-0.35-SCM	54.54 *0.11*	69.33 *1.04*	80.58 *0.08*	87.63 *2.80*	91.92 *0.38*
20FA-0.35-N	35.13 *0.94*	41.96 *0.43*	55.45 *0.26*	60.29 *0.18*	73.04 *0.64*
20FA-0.35-H	39.40 *0.68*	53.45 *0.38*	63.49 *0.80*	72.60 *0.24*	87.56 *0.39*
20FA-0.35-SCM	40.10 *1.07*	54.44 *0.24*	65.16 *0.12*	75.67 *0.41*	90.40 *0.30*
40FA-0.35-N	17.11 *0.27*	32.66 *0.18*	39.52 *1.26*	53.70 *0.76*	72.69 *0.94*
40FA-0.35-H	22.99 *0.26*	36.11 *0.65*	46.48 *0.33*	58.23 *0.31*	77.47 *1.04*
40FA-0.35-SCM	25.66 *0.80*	41.17 *0.23*	52.47 *0.36*	62.31 *0.12*	82.27 *0.27*
60FA-0.35-N	17.99 *0.12*	28.05 *0.58*	33.41 *1.15*	42.51 *2.05*	58.70 *0.31*
60FA-0.35-H	19.01 *0.34*	29.09 *0.71*	38.31 *0.60*	47.26 *0.44*	61.70 *1.06*
60FA-0.35-SCM	22.20 *0.18*	32.28 *0.46*	40.83 *1.03*	50.63 *0.60*	63.62 *0.24*

***** Standard deviation within ±3% is indicated in italics.

**Table A2:** Compressive strength for w/b 0.40.

Mixture	Compressive Strength (MPa) *				
	3 Days	7 Days	14 Days	28 Days	90 Days
C-0.40-N	44.63 *1.59*	59.94 *1.79*	62.87 *1.91*	66.45 *0.39*	75.50 *0.87*
C-0.40-H	47.84 *0.29*	62.19 *0.13*	65.23 *0.10*	71.69 *0.62*	78.66 *0.21*
C-0.40-SCM	50.82 *1.26*	64.66 *1.58*	67.08 *0.77*	73.80 *2.72*	82.73 *1.87*
10FA-0.40-N	42.02 *0.53*	48.21 *0.70*	52.93 *0.40*	61.24 *1.79*	70.29 *0.94*
10FA-0.40-H	45.58 *0.41*	50.43 *0.51*	57.67 *2.10*	66.72 *2.72*	74.03 *0.26*
10FA-0.40-SCM	47.83 *0.39*	52.36 *0.34*	59.26 *1.41*	68.84 *0.14*	77.77 *1.21*
20FA-0.40-N	29.61 *1.07*	37.37 *1.05*	54.27 *0.80*	57.22 *1.91*	66.27 *0.45*
20FA-0.40-H	32.46 *0.13*	40.04 *2.18*	58.63 *1.46*	62.86 *0.81*	70.45 *0.91*
20FA-0.40-SCM	35.30 *0.85*	42.71 *0.45*	60.06 *0.75*	65.69 *0.20*	74.62 *1.18*
40FA-0.40-N	18.27 *0.60*	35.92 *0.58*	48.48 *1.75*	57.05 *0.11*	66.10 *0.43*
40FA-0.40-H	19.78 *0.38*	37.16 *1.14*	52.34 *0.71*	61.19 *1.31*	69.21 *0.69*
40FA-0.40-SCM	24.62 *0.61*	40.21 *0.72*	55.73 *1.12*	63.38 *1.34*	72.31 *1.07*
60FA-0.40-N	15.67 *0.44*	18.46 *0.94*	24.46 *1.17*	29.52 *2.05*	38.57 *0.96*
60FA-0.40-H	16.82 *0.77*	20.38 *0.41*	26.74 *1.18*	32.42 *0.93*	41.69 *1.08*
60FA-0.40-SCM	18.14 *1.24*	23.07 *1.79*	28.32 *0.62*	35.87 *1.41*	44.80 *0.95*

***** Standard deviation within ±3% is indicated in italics.

**Table A3:** Compressive strength for w/b 0.45.

Mixture	Compressive Strength (MPa) *				
	3 Days	7 Days	14 Days	28 Days	90 Days
C-0.45-N	30.74 *0.77*	41.93 *0.67*	49.17 *1.40*	56.93 *0.81*	65.98 *0.92*
C-0.45-H	34.79 *0.63*	45.82 *0.89*	54.43 *0.17*	61.72 *1.67*	69.73 *0.42*
C-0.45-SCM	40.76 *0.68*	48.95 *0.16*	57.28 *0.81*	64.43 *1.85*	73.48 *1.17*
10FA-0.45-N	31.77 *2.41*	43.44 *0.81*	50.50 *0.40*	58.08 *1.15*	67.13 *0.94*
10FA-0.45-H	33.68 *0.25*	46.45 *1.27*	53.45 *0.20*	60.51 *1.54*	69.33 *1.27*
10FA-0.45-SCM	35.83 *0.95*	49.26 *0.39*	56.17 *0.85*	62.48 *0.67*	71.53 *1.31*
20FA-0.45-N	21.33 *0.16*	29.21 *0.55*	35.88 *0.69*	43.24 *0.10*	53.29 *1.14*
20FA-0.45-H	23.73 *0.47*	32.64 *1.40*	40.51 *0.32*	49.11 *1.37*	56.98 *0.87*
20FA-0.45-SCM	29.21 *0.75*	35.07 *0.29*	42.77 *1.83*	52.62 *0.58*	61.67 *1.24*
40FA-0.45-N	14.25 *0.26*	21.68 *0.34*	27.11 *0.32*	31.42 1.16	41.47 *0.83*
40FA-0.45-H	16.50 *0.50*	22.96 *0.31*	28.96 *2.01*	33.71 *0.77*	44.18 *1.23*
40FA-0.45-SCM	18.56 *0.23*	25.39 *1.25*	30.69 *0.37*	36.83 *1.43*	46.88 *0.87*
60FA-0.45-N	13.18 *1.15*	19.78 *0.93*	26.17 *0.82*	30.87 *1.34*	40.24 *0.52*
60FA-0.45-H	15.65 *0.78*	21.29 *0.64*	27.82 *1.04*	32.19 *0.90*	42.78 *0.89*
60FA-0.45-SCM	17.24 *1.39*	24.61 *0.75*	29.94 *0.44*	35.61 *0.32*	45.31 *0.59*

***** Standard deviation within ±3% is indicated in italics.

**Table A4:** Compressive strength for w/b 0.50.

Mixture	Compressive Strength (MPa) *				
	3 Days	7 Days	14 Days	28 Days	90 Days
C-0.50-N	18.27 *0.73*	25.18 *0.25*	30.47 *1.33*	38.47 *0.14*	51.02 *1.07*
C-0.50-H	20.41 *0.58*	28.23 *0.92*	35.73 *0.29*	42.87 *1.41*	54.63 *1.12*
C-0.50-SCM	23.92 *0.29*	34.21 *0.72*	41.90 *1.41*	50.19 *0.09*	58.24 *1.05*
10FA-0.50-N	16.87 *0.61*	23.62 *0.39*	28.47 *1.33*	36.71 *0.77*	46.76 *1.07*
10FA-0.50-H	18.29 *0.68*	26.73 *0.54*	31.86 *0.93*	41.25 *1.03*	51.30 *1.57*
10FA-0.50-SCM	21.14 *0.09*	29.84 *0.16*	35.24 *0.19*	45.78 *0.15*	55.83 *1.14*
20FA-0.50-N	15.23 *0.67*	23.02 *0.13*	27.96 *0.75*	35.14 *1.12*	45.39 *1.09*
20FA-0.50-H	18.02 *0.32*	25.72 *0.65*	31.43 *0.42*	39.21 *0.67*	49.59 *0.35*
20FA-0.50-SCM	20.81 *0.89*	28.42 *0.43*	34.89 *1.98*	43.27 *0.86*	53.87 *0.64*
40FA-0.50-N	14.97 *0.22*	22.86 *0.56*	26.74 *0.21*	34.95 *0.45*	44.23 *1.69*
40FA-0.50-H	17.57 *0.98*	25.39 *1.78*	30.33 *0.67*	38.57 *1.02*	47.96 *0.93*
40FA-0.50-SCM	20.17 *0.34*	27.92 *1.67*	33.91 *0.39*	42.18 *0.82*	51.68 *0.74*
60FA-0.50-N	14.12 *0.92*	20.72 *0.41*	26.07 *1.21*	33.87 *1.08*	43.14 *0.71*
60FA-0.50-H	17.03 *0.86*	23.11 *0.79*	29.44 *0.29*	37.05 *0.69*	46.55 *1.49*
60FA-0.50-SCM	19.94 *0.26*	25.49 *0.64*	32.81 *0.94*	40.23 *1.09*	49.95 *0.78*

***** Standard deviation within ±3% is indicated in italics.
